# Evidence of BK Polyomavirus Infection in Urothelial but not Renal Tumors from a Single Center Cohort of Kidney Transplant Recipients

**DOI:** 10.3390/v13010056

**Published:** 2021-01-03

**Authors:** Cinzia Borgogna, Silvia Albertini, Licia Martuscelli, Filippo Poletti, Alessandro Volpe, Guido Merlotti, Vicenzo Cantaluppi, Renzo Boldorini, Marisa Gariglio

**Affiliations:** 1 Virology Unit, Department of Translational Medicine, Novara Medical School, 28100 Novara, Italy; cinzia.borgogna@med.uniupo.it (C.B.); silvia.albertini@med.uniupo.it (S.A.); licia.martuscelli@med.uniupo.it (L.M.); 2 Urology Unit, Department of Translational Medicine, Novara Medical School, 28100 Novara, Italy; filippopoletti91@gmail.com (F.P.); alessandro.volpe@med.uniupo.it (A.V.); 3 Nephrology and Kidney Transplantation Unit, Department of Translational Medicine, Novara Medical School, 28100 Novara, Italy; gmerlotti87@gmail.com (G.M.); vincenzo.cantaluppi@med.uniupo.it (V.C.); 4 Pathology Unit, Department of Health Sciences, Novara Medical School, 28100 Novara, Italy; renzo.boldorini@med.uniupo.it

**Keywords:** BKPyV, PyVAN, kidney transplant recipient, renal cell carcinoma, bladder carcinoma

## Abstract

Emerging evidence indicates that reactivation of BK polyomavirus (BKPyV) in the kidney and urothelial tract of kidney transplant recipients (KTRs) may be associated with cancer in these sites. In this retrospective study of a single center cohort of KTRs (*n* = 1307), 10 clear cell renal cell carcinomas and 5 urinary bladder carcinomas were analyzed from 15 KTRs for the presence of BKPyV infection through immunohistochemistry and fluorescent in situ hybridization (FISH). Three of these patients had already exhibited biopsy-proven polyomavirus-associated nephropathies (PyVAN). Although the presence of BKPyV large-T antigen was evident in the urothelium from a kidney removed soon after PyVAN diagnosis, it was undetectable in all the formalin-fixed and paraffin-embedded (FFPE) blocks obtained from the 10 kidney tumors. By contrast, large-T antigen (LT) labeling of tumor cells was detected in two out of five bladder carcinomas. Lastly, the proportion of BKPyV DNA-FISH-positive bladder carcinoma nuclei was much lower than that of LT-positive cells. Taken together, our findings further strengthen the association between BKPyV reactivation and cancer development in KTRs, especially bladder carcinoma.

## 1. Introduction

Immunosuppressive treatment of kidney transplant recipients (KTRs) is a well-known risk factor for infectious diseases and their complications, including infection-related malignancies [1,2,3,4]. A causal link has been established between the following tumors and viral infections, as examples: (i) anogenital cancer and human papillomavirus (HPV); (ii) Merkel cell carcinoma and Merkel cell polyomavirus (MCPyV); (iii) immune suppression-related non-Hodgkin lymphoma and Epstein–Barr virus (EBV); and (iv) Kaposi’s sarcoma and KS herpesvirus (KSHV/HHV8) [5,6,7,8,9]. In addition, a yet to be confirmed association between human polyomavirus (HPyV), especially BK polyomavirus (BKPyV), infection/reactivation with renal (native) or urinary tract (ureter and urinary bladder) carcinoma development has been proposed [5,9,10,11,12,13,14].

HPyVs are small non-enveloped icosahedral particles with circular double-stranded DNA genomes of ≈5 kb, divided into three regions: the early region, which encodes the large-T and small-t antigens (LT and sT, respectively); the late region, which encodes the virion structural proteins (VP1, VP2); and the control region, which encompasses the origin of replication and transcription regulatory elements. BKPyV can cause lytic infection of renal tubule cells, resulting in the loss of these cells. Furthermore, uncontrolled BKPyV infection contributes to polyomavirus-associated nephropathy (PyVAN) in KTRs [15,16,17]. A growing body of evidence also suggests a correlation between BKPyV infection and urothelial carcinoma in KTRs, affecting either the transplanted organ or the urinary tract of the recipient [18,19]. 

The central oncogenesis mechanism of HPyV involves disruption of the tumor suppressor genes p53 and pRb, mediated by the early viral gene products, including the LT [15,18]. In addition, emerging evidence indicates that BKPyV LT can promote tumor formation, in part through the upregulation of members belonging to the APOBEC3 family of cytosine deaminases [20]. 

In this long-term retrospective study of a single center cohort of KTRs, we looked for evidence of HPyV infection in urinary tract and renal tumors by means of immunostaining and fluorescent in situ hybridization (FISH). Our aim was to determine the extent of HPyV reactivation in the anatomical sites where these tumors had arisen in order to establish a potential association between the ubiquitous virus reactivation in the context of long-lasting iatrogenic immunosuppression and cancer development.

## 2. Materials and Methods 

### 2.1. Study Design, Inclusion Criteria, and Samples

This study was a retrospective analysis of prospectively collected data obtained from patients from a single Kidney Transplant Center at the University Hospital in Novara, starting from its opening in November 1998 to June 2020. The study population included adult patients undergoing first or subsequent living- or deceased-donor kidney transplantation (KTx). No patient received simultaneous combined organ transplantations. A strict pre-transplant screening for malignancy and pre-malignant lesions was performed at our center in order to exclude patients with active neoplasia, in accordance with local and national guidelines. 

All patients were informed at the time of transplantation or surgery that their clinical data would be used for research purposes, and all patients signed a written informed consent form. The study approval was obtained from the Ethics Committee of “Maggiore della Carità” Hospital, ASL BI, ASL NO, ASL VCO Protocol 1037/CE, Study No. CE 169/16.

Tissue sections were obtained from formalin-fixed and paraffin-embedded (FFPE) blocks, previously collected and stored in the University Hospital medical material archives.

### 2.2. Immunohistochemistry and Fluorescent in Situ Hybridization (FISH)

Consecutive 5 μm sections obtained from FFPE tissues were processed for immunohistochemistry using the automated immunostainer BenchMark ULTRA Stainer (Ventana Medical System, Tucson, AZ, USA) using the anti-Large T SV40 (clone MRQ-4) and anti-p16^INK4a^ (clone E6H4) (Ventana Medical System, Tucson, AZ, USA). LT-positive cases were further tested by DNA-fluorescent in situ hybridization (FISH), using a probe derived from nick translation (Biotin Nick Translation Mix, Roche, Basel, CH, USA) of the plasmid containing the entire BKPyV type I (Dunlop) genome (ATCC number 45025). Briefly, tissue sections were dewaxed and heated in a pressure cooker in antigen retrieval buffer (Vector Laboratories, Burlingame, CA, USA) at 750W for 15 min, followed by an additional step at 350W for 10 min. Probe and target DNA were denatured simultaneously for 5 min at 90 °C prior to hybridization. BKPyV probes were detected through tyramide signal amplification, according to the manufacturer’s instructions (PerkinElmer Life and Analytical Sciences Inc., Shelton, CT, USA). Images were acquired using a digital scanner (Pannoramic MIDI; 3D Histech Kft., Budapest, Hungary). 

## 3. Results and Discussion

A total of 1307 KTxs were performed in 1288 patients (aged 51.3 ± 12.5 years; 63.6% males). Transplanted kidneys were mainly obtained from deceased donors (94.2%). During a mean follow-up period of 8.45 ± 3.9 years after KTx, the cumulative malignancy incidence was 0.4%, 0.9%, and 1.3% at 1, 5, and 15 years after transplant, respectively. The mean time from transplant to the primary lesion of the kidney or urinary tract was 4 ± 4.3 years.

**Figure 1:**
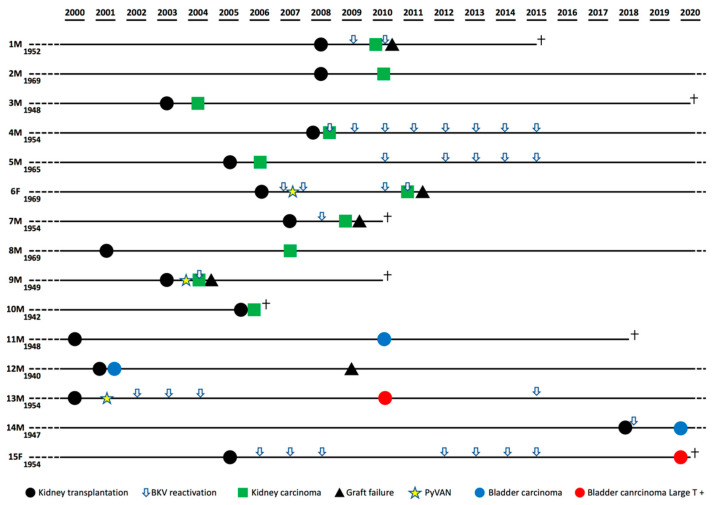
Patients’ clinical history timelines highlighting BK polyomavirus (BKPyV) reactivation.

In our study cohort, 15 urinary tract malignancies or clear cell renal cell carcinomas (ccRCCs) arose in 15 KTRs. As reported in Table 1, they comprised eight ccRCCs from native kidneys, two ccRCCs from transplanted kidneys, and five carcinomas from the bladder. Unfortunately, data for BKPyV viremia were only available for nine of the study participants and revealed that eight of them had documented viremia at the time of cancer diagnosis, while both LT-positive bladder cancer patients displayed a clinical history characterized by numerous episodes of BKPyV reactivation over an extended period of time (Figure 1). Three of these patients (3/15; 20%) had already exhibited PyVAN, whereas no patient had presented with PyVAN after cancer development. Interestingly, the percentage of urinary tract cancer patients with a previous diagnosis of PyVAN (3/16, total number of biopsy-proven PyVAN cases in the study cohort; 18.75%) was significantly higher than the percentage of KTRs with no history of PyVAN (12/1285; 0.93%). 

Next, tissue sections from the aforementioned surgical specimens, as well as surrounding normal tissues, were immunolabeled with anti-LT antibodies (pan-polyomavirus antigen), and those found to be LT-positive were also processed by FISH analysis using the BKPyV genome as a probe. 

**Figure 2:**
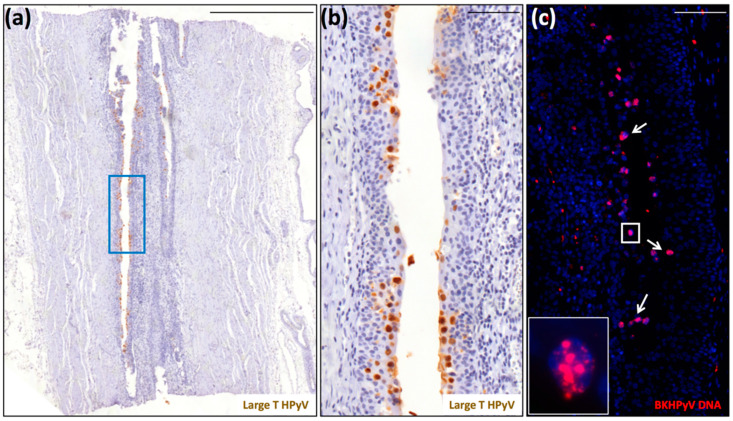
Detection of polyomavirus large-T antigen and BKPyV DNA in the ureter of a native kidney removed from patient 9 due to the presence of a clear cell renal cell carcinoma. (**a**) Scan of a tissue section stained for large-T antigen (scale bar: 500 μm). (**b**) Region corresponding to the blue square highlighted in panel (a) (scale bar: 100 μm). This section was counterstained with hematoxylin to visualize cell nuclei. (**c**) Serial section stained for BKPyV genome by fluorescent in situ hybridization (FISH) (red) (inset: magnification of the white square). The white arrows indicate FISH-BKPyV-positive nuclei. This section was counterstained with 4′,6-diamidino-2-phenylindole (DAPI) (blue) to visualize cell nuclei (scale bar: 100 μm).

All the ccRCCs resulted negative for LT expression in tumor cells (data not shown). LT labeling was only detected in the urothelium (Figure 2a,b) from patient 9. In addition, the FISH signal in these cells was quite strong and diffused, with the presence of large dots, very much resembling sites of intense viral genome replication (Figure 2c). Although these finding may not be surprising given the patient’s very recent diagnosis of PyVAN in the kidney graft 3 months earlier, they appear to be biologically relevant as they show massive BKPyV infection in both kidneys. Importantly, even though the virus was no longer detectable in the tumor cells, it is likely that this earlier infection might have contributed to virus-mediated tumorigenesis, a hypothesis supported by our previous findings showing that PyVAN in patients 6 and 9—namely, patient 2 and 1 in Peretti et al. [21]—was associated with donor-derived de novo infections with BKV-Ib2 or BKV-IV that, during the development of nephropathy, acquired mutations to elude the host immune response. Indeed, the dominant VP1 mutations detected in both patients were consistent with DNA damage induced by APOBEC3B, a cytidine deaminase upregulated following infection with various RNA and DNA viruses. This would also be consistent with several reports showing that BKPyV infection can specifically induce APOBEC3B in vitro and in vivo, thereby leading to genome instability and eventually cancer development [20,22,23].

Two of the five urinary bladder carcinomas showed positive LT labeling. Patient 13 had developed documented PyVAN nine years prior to the onset of bladder carcinoma, and BKPyV reactivation remained detectable as viremia before and after cancer surgery. The clinical history of patient 15 was remarkable as she had experienced repeated episodes of BKPyV viremia over the course of 15 years, from transplant to cancer surgery. 

**Figure 3:**
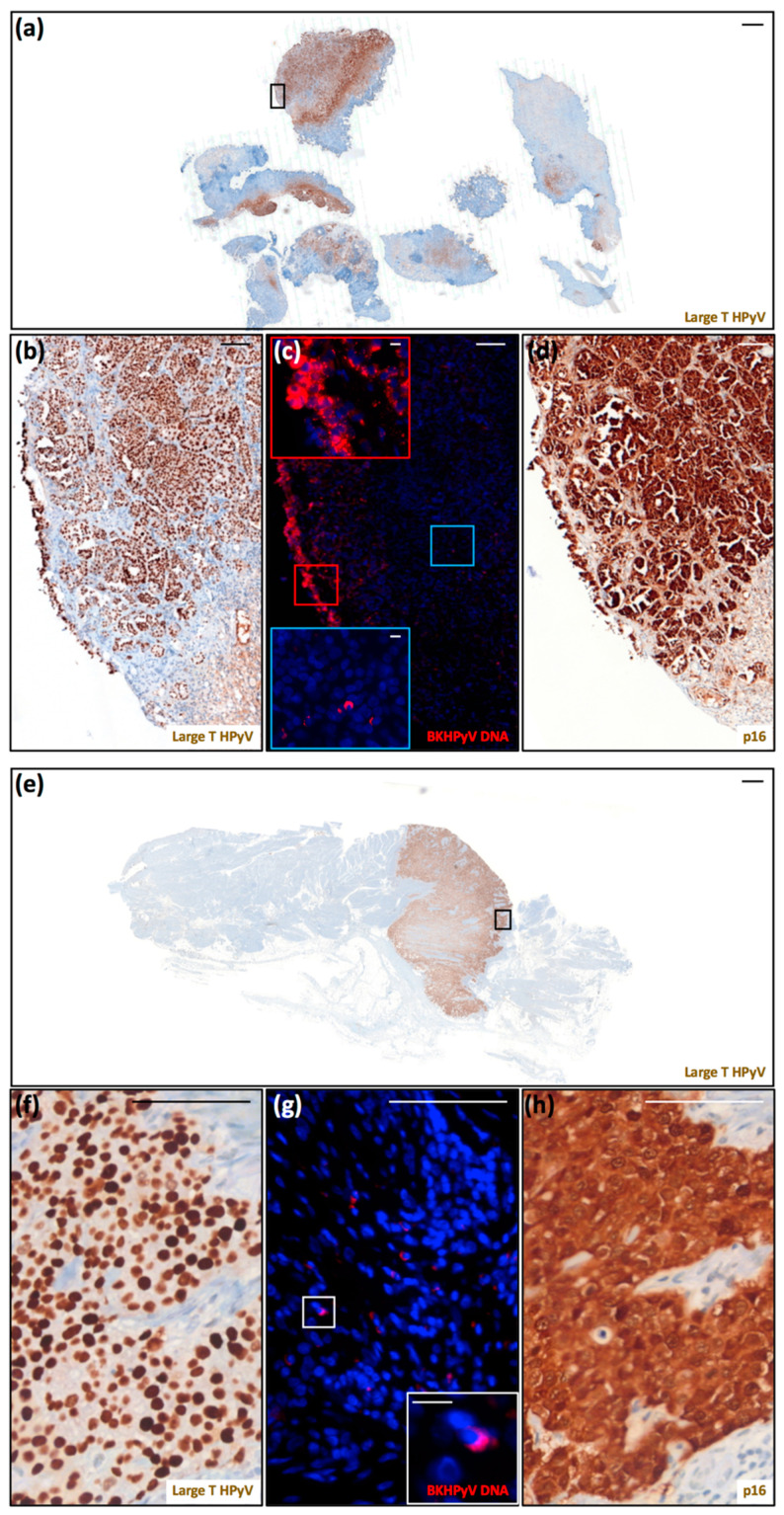
Detection of polyomavirus large-T antigen, BKPyV DNA, and cellular p16^INK4a^ in two urinary bladder carcinomas. (**a**) Scan of a tissue section of the urinary bladder carcinoma from patient 13 labeled for LT (scale bar: 1000 μm). (**b**–**d**) These panels correspond to the black square highlighted in (**a**). (**b**) Magnification of LT labeling (scale bar: 100 μm). (**d**) Scan of a serial section labeled for p16^INK4a^ (scale bar: 100 μm). These sections were counterstained with hematoxylin to visualize cell nuclei. (**c**) Serial section stained for BKPyV genome by FISH (red) (scale bar: 100 μm). The regions shown in the insets correspond to the red (superficial) and blue (internal) squares (scale bar: 10 μm). This section was counterstained with 4′,6-diamidino-2-phenylindole (DAPI) (blue) to visualize cell nuclei. (**e**) Scan of a tissue section of the urinary bladder carcinoma from patient 15 stained for LT (scale bar: 1000 μm). (**f**–**h**) These panels correspond to the black square highlighted in (**e**). (**f**) Magnification of LT labeling (scale bar: 100 μm). (**h**) Scan of a serial section stained for p16^INK4a^ (scale bar: 100 μm). These sections were counterstained with hematoxylin to visualize cell nuclei. (**g**) Serial section stained for BKPyV genome by FISH (red) (scale bar: 100 μm). The regions shown in the insets correspond to the white square (scale bar: 10 μm). This section was counterstained with 4′,6-diamidino-2-phenylindole (DAPI) (blue) to visualize cell nuclei.

As shown in Figure 3, the majority of the tumor cells from these two invasive high-grade transitional carcinomas displayed very strong LT labeling. Consistent with LT being overexpressed in dysplastic cells, we were also able to detect sustained p16^INK4a^ expression in both carcinomas. The same labeling pattern for both LT and p16^INK4a^ expression was also observed in tissue sections from the transurethral resection of the bladder tumor (TURBT), performed before cystectomy (data not shown). Some LT-positive cells were also found in the urethra, very likely shed from the infected urothelium (data not shown). In the tumor from patient 13, the nuclear FISH signal was mostly restricted to superficial/external urothelial cells (Figure 3c, red square vs. internal blue square), where viral genome amplification was taking place. In the tumor from patient 15, several clusters of cells with nuclear FISH signal were randomly distributed throughout the tumor (Figure 3g). The uniform LT labeling of these two bladder carcinomas, similar to that reported by previous studies [18,24,25,26,27], suggests that deregulated LT expression may be associated with the transformation process. Consistent with the fact that in tumor cells virus replication is supposed to be quite low, the proportion of cells with FISH signal was significantly lower than that of cells displaying LT labeling. Altogether, our observations strengthen the notion that BKPyV may contribute to malignancies in its respective sites of infection, implicating the need for further investigations into this potential cancer-causing factor in KTRs. 

## Figures and Tables

**Table 1:** Clinical history of patients developing kidney and urinary tract cancer.

Patient	Birth Year	KTx Date	Tumor Type and Grade	Pathological Stage	PyVAN	Notes
***1M***	1952	2008	2010: Clear cell RCC FG 2	T1a	No	2010: Graft failure /second transplant 2010: Acute promyelocytic leukemia 2015: Death
***2M***	1969	2008	2010: Clear cell RCC FG 3	T1b	No	
***3M***	1948	2003	2004: Clear cell RCC FG 2	T1a	No	2020: Death
***4M***	1954	2008	2008: Papillary RCC type 1 FG 2	T1a	No	
***5M***	1965	2005	2006: Clear cell RCC FG 3	T1a	No	2011: Acute HBV infection
***6F***	1969	2006	2011: Clear cell RCC FG 4 (graft)	T3b N2	2007	2007 and 2011: CIN 2011: Graft failure
***7M***	1954	2007	2009: Clear cell RCC FG 2	T3a	No	2009: Graft failure 2010: Death
***8M***	1969	2001	2007: Clear cell RCC FG 2 (graft)	T1a M1	No	2006: Viral enteritis
***9M***	1949	2003	2004: Clear cell RCC FG 2	T1a	2004	2004: Graft failure 2010: Death
***10M***	1942	2006	2006: Clear cell RCC FG 2	T1a	No	2006: Pneumonia and CMV reactivation 2006: Death
***11M***	1948	2000	2010: LG urothelial carcinoma	Ta	No	2001: CMV disease (hematological) 2002: Skin cancer and VZV reactivation 2006: Skin cancer 2007: MGUS IgG-k (plasma cells at BMB: 5%) 2014: Sepsis 2018: Death
***12M***	1940	2001	2001: HG In situ urothelial carcinoma		No	2005: Prostatic cancer (Gleason 6) 2009: Graft failure
***13M***	1954	2000	2010: LG Urothelial carcinoma	T1	2001	
***14M***	1947	2018	2020: HG Urothelial carcinoma		No	
***15F***	1954	2005	2020: HG Urothelial carcinoma	T3a N2	No	2020: Death

KTx: Kidney transplantation; RCC: renal cell carcinoma; FG: Fuhrmann grade; CIN: cervical intraepithelial neoplasia; PyVAN: polyomavirus-associated nephropathy; HBV: hepatitis B virus; CMV: cytomegalovirus; SCC: squamous cell carcinoma; AK: actinic keratosis; VZV: varicella zoster virus; LG: low-grade; MGUS: monoclonal gammopathy of undetermined significance; BMB: bone marrow biopsy; HG: high-grade; M: male; F: female.

## Data Availability

The data presented in this study are available on request from the corresponding author.

## References

[1] Vajdic C.M., Van Leeuwen M.T. (2009). Cancer incidence and risk factors after solid organ transplantation. Int. J. Cancer.

[2] Sherston S.N., Carroll R.P., Harden P.N., Wood K.J. (2014). Predictors of Cancer Risk in the Long-Term Solid-Organ Transplant Recipient. Transplantation.

[3] Wątorek E., Boratyńska M., Smolska D., Patrzalek D., Klinger M. (2011). Malignancy after renal transplantation in the new era of immunosuppression. Ann. Transplant..

[4] Schulz T.F. (2009). Cancer and viral infections in immunocompromised individuals. Int. J. Cancer.

[5] Geissler E.K. (2015). Post-transplantation malignancies: Here today, gone tomorrow?. Nat. Rev. Clin. Oncol..

[6] Grulich A.E., Van Leeuwen M.T., Falster M.O., Vajdic C.M. (2007). Incidence of cancers in people with HIV/AIDS compared with immunosuppressed transplant recipients: A meta-analysis. Lancet.

[7] Hall E.C., Pfeiffer R.M., Segev D.L., Engels E.A. (2013). Cumulative incidence of cancer after solid organ transplantation. Cancer.

[8] Ettorre G.M., Piselli P., Galatioto L., Rendina M., Nudo F., Sforza D., Miglioresi L., Fantola G., Cimaglia C., Vennarecci G. (2013). De Novo Malignancies Following Liver Transplantation: Results From a Multicentric Study in Central and Southern Italy, 1990–2008. Transplant. Proc..

[9] Piselli P., Serraino D., Segoloni G.P., Sandrini S., Piredda G.B., Scolari M.P., Rigotti P., Busnach G., Messa P., Donati D. (2013). Risk of de novo cancers after transplantation: Results from a cohort of 7217 kidney transplant recipients, Italy 1997–2009. Eur. J. Cancer.

[10] Madeleine M.M., Finch J.L., Lynch C.F., Goodman M.T., Engels E.A. (2013). HPV-Related Cancers After Solid Organ Transplantation in the United States. Arab. Archaeol. Epigr..

[11] Madeleine M.M., Johnson L.G., Daling J.R., Schwartz S.M., Carter J.J., Berg D., Nelson K., Davis C.L., Galloway D.A. (2012). Cohort profile: The skin cancer after organ transplant study. Int. J. Epidemiol..

[12] Einollahi B., Simforoosh N., Lessan-Pezeshki M., Basiri A., Nafar M., Gholi F.P.-R., Firouzan A., Ahmadpour P., Makhdomi K., Ghafari A. (2009). Genitourinary Tumor Following Kidney Transplantation: A Multicenter Study. Transplant. Proc..

[13] Proby C., Harwood C.A., Neale R.E., Green A.C., Euvrard S., Naldi L., Tessari G., Feltkamp M.C.W., De Koning M.N.C., Quint W.G.V. (2011). A Case-Control Study of Betapapillomavirus Infection and Cutaneous Squamous Cell Carcinoma in Organ Transplant Recipients. Arab. Archaeol. Epigr..

[14] Jung J.-W., Overgaard N.H., Burke M.M.T., Isbel N., Frazer I.H., Simpson F., Wells J.W. (2015). Does the nature of residual immune function explain the differential risk of non-melanoma skin cancer development in immunosuppressed organ transplant recipients?. Int. J. Cancer.

[15] DeCaprio J.A., Garcea R.L. (2013). A cornucopia of human polyomaviruses. Nat. Rev. Genet..

[16] Dalianis T., Hirsch H.H. (2013). Human polyomaviruses in disease and cancer. Virology.

[17] Nickeleit V., Singh H.K. (2015). Polyomaviruses and disease. Curr. Opin. Organ Transplant..

[18] Papadimitriou J.C., Randhawa P., Rinaldo C.H., Drachenberg C.B., Alexiev B.A., Hirsch H.H. (2016). BK Polyomavirus Infection and Renourinary Tumorigenesis. Arab. Archaeol. Epigr..

[19] Alexiev B.A., Drachenberg C., Papadimitriou J.C. (2015). Polyomavirus-Cystitis Associated With In Situ and Invasive Urothelial Carcinoma in a Heart Transplant Recipient. Transplantion.

[20] Verhalen B., Starrett G.J., Harris R.S., Jiang M. (2016). Functional Upregulation of the DNA Cytosine Deaminase APOBEC3B by Polyomaviruses. J. Virol..

[21] Peretti A., Geoghegan E.M., Pastrana D.V., Smola S., Feld P., Sauter M., Lohse S., Ramesh M., Lim E.S., Wang D. (2018). Characterization of BK Polyomaviruses from Kidney Transplant Recipients Suggests a Role for APOBEC3 in Driving In-Host Virus Evolution. Cell Host Microbe.

[22] Roelofs P.A., Goh C.Y., Chua B.H., Jarvis M.C., Stewart T.A., McCann J.L., McDougle R.M., Carpenter M.A., Martens J.W.M., Span P.N. (2020). Characterization of the mechanism by which the RB/E2F pathway controls expression of the cancer genomic DNA deaminase APOBEC3B. eLife.

[23] Starrett G.J., Serebrenik A.A., Roelofs P.A., McCann J.L., Verhalen B., Jarvis M.C., Stewart T.A., Law E.K., Krupp A., Jiang M. (2019). Polyomavirus T Antigen InducesAPOBEC3BExpression Using an LXCXE-Dependent and TP53-Independent Mechanism. mBio.

[24] Kuppachi S., Holanda D., Michael E., Tyler A.J., Wissel M.C., Kleiboeker S.B., Alexiev B., Thomas C.P. (2016). An Unexpected Surge in Plasma BKPyV Viral Load Heralds the Development of BKPyV-Associated Metastatic Bladder Cancer in a Lung Transplant Recipient With BKPyV Nephropathy. Arab. Archaeol. Epigr..

[25] Bialasiewicz S., Cho Y., Rockett R., Preston J., Wood S., Fleming S., Shepherd B., Barraclough K., Sloots T., Isbel N. (2013). Association of micropapillary urothelial carcinoma of the bladder and BK viruria in kidney transplant recipients. Transpl. Infect. Dis..

[26] Alexiev B.A., Papadimitriou J.C., Chai T.C., Ramos E., Staats P.N., Drachenberg C. (2013). Polyomavirus (BK)-associated pleomorphic giant cell carcinoma of the urinary bladder: A case report. Pathol. Res. Pr..

[27] Roberts I.S.D., Besarani D., Mason P., Turner G., Friend P.J., Newton R. (2008). Polyoma virus infection and urothelial carcinoma of the bladder following renal transplantation. Br. J. Cancer.

